# Stronger together: How unicellular algae respond to stress by socialization

**DOI:** 10.1093/plphys/kiac398

**Published:** 2022-08-25

**Authors:** Bernarda Calla

**Affiliations:** United States Department of Agriculture-Agricultural Research Service (USDA-ARS), Forage Seed and Cereal Research Unit, Corvallis, Oregon, USA

Social behaviour is defined as the interaction between two or more individuals of the same species and includes “all behavior that tends to bring individuals together” (Whishaw et al., 2006). Unicellular organisms can be highly social, frequently forming structured groups and expressing collective behaviour. For example, bacteria are capable of quorum sensing, a mechanism by which cells communicate to sense population density (Miller and Bassler, 2001). In a more unique example, the Gram-negative bacteria *Myxococcus xanthus* expresses cooperative feeding by hunting for food in swarms and forms fruiting bodies upon starvation (Dworkin and Kaiser, 1985). Microorganisms can also socialize by forming cell aggregates, such as biofilms attached to surfaces (Stoodley et al., 2002) or surface-free aggregates (Cai, 2020) in response to changes in environmental conditions.

Among eukaryotic organisms, unicellular algae also form cell aggregates. The unicellular green alga Chlamydomonas (*Chlamydomonas reinhardtii*), an important model for the study of modern photosynthetic organisms and the evolution of land plants, forms aggregates in response to factors such as predation, changes in pH, exposure to chemicals, and nutritional needs (Herron et al., 2019). Chlamydomonas can form several different types of aggregates. Palmelloids, for example, are small groups of 4–16 cells that form in response to exposure to organic acids (Iwasa and Murakami, 1968) and are the product of incomplete clonal divisions. Other small Chlamydomonas cell aggregates have also been described, including gloeocapsoids that resemble the structures of cyanobacteria of the genera *Gloeocapsa* and *Gloeothece* (Krespach et al., 2021). Larger Chlamydomonas cell aggregates consisting of ten to more than thousands of cells have also been identified after exposure to some abiotic stressors. These larger aggregates differ from palmelloids in that they form from differentiated cells that can be genetically heterogeneous (Sathe and Durand, 2016).

In this issue of *Plant Physiology*, de Carpentier et al. (2022) use reverse genetics together with a multiomics approach to determine the genetic processes behind large cell aggregation in Chlamydomonas responding to abiotic stress. The authors constructed a library of insertional mutants and identified six Chlamydomonas mutant lines with the ability to spontaneously form cell aggregates: the *socializer* (*saz*) mutants. The *saz1* mutant locus was in the Cre01.g049950 gene that encodes the vegetative lytic enzyme. By growing cultures of *saz1* with a Chlamydomonas line expressing a yellow fluorescence protein (mVenus), the authors demonstrated that the aggregates formed can be genetically heterogeneous, as opposed to clonally formed (i.e. cells of both the *saz1* mutant and the mVenus line were found together in a single aggregate). The *saz1* mutants were more tolerant to abiotic stresses, including heat shock, and an extracellular matrix resembling that of bacterial biofilms was readily visible surrounding the aggregates.

To identify the specific genes and proteins that underlie aggregation behaviour, the authors tested the media in which the *saz1* mutants had been growing. The authors reported that media isolated from actively growing *saz1* cultures, from which all living cells had been removed, were capable of inducing aggregation in naïve wild-type (WT) cells, indicating that the factors that promote aggregation are secreted into the media.

A quantitative proteomics analysis identified 131 proteins differentially present in the media in which different *saz* mutants had grown compared to the media in which WT Chlamydomonas had grown. The major disparities in protein composition detected between the *saz1* and the WT media consisted of different levels of pherophorins (PHCs), matrix metalloproteinases (MMPs), vegetative serine–proline-rich proteins (VSPs), and lysis oxidases. Transcriptomic analyses of six of the *saz* mutants complemented the proteomic analyses, confirming that proteins in the families of pherophorins, proteases, and lipases differ between the *saz* mutants and WT Chlamydomonas. Transcript levels of these differentially expressed proteins corresponded to the protein levels in the media about one-third of the time, suggesting that some posttranscriptional regulation is involved in the expression of these secreted aggregation-associated proteins.

The authors also obtained *phc, mms, and vsp* mutants from the *Chlamydomonas reinhardtii* Library Project (Li et al., 2019) and confirmed that three pherophorin mutants (*phc30*, *phc41*, and *phc50*) do not aggregate in response to abiotic stress, indicating that these particular pherophorins promote aggregation. Conversely, two other pherophorin mutants (*phc28*and *phc35*), one MMP mutant (*mmp32*) and the other *vsp4* mutant spontaneously formed aggregates, indicating the normal protein products of these genes inhibit aggregation.

Several authors have proposed microalgal cell aggregation and other unicellular social behaviour as a possible evolutionary step toward multicellularity (Ratcliff et al., 2013; Sathe and Durand, 2016; Krespach et al., 2021). The work by de Carpentier et al. (2022) contributes valuable insight regarding the mechanisms of large cell aggregation in Chlamydomonas, including the identification of pro- and anti-aggregation genes that underpin the process of aggregation. This study opens doors for further understanding the evolution of both multicellularity and primitive social interactions.

## Disclaimer

The author contributed to this article in her personal capacity. The views and opinions expressed within are those of the author and do not necessarily represent the views of the Agricultural Research Service, USDA, or the US Government.

*Conflict of interest statement*. None declared.
